# New Pyridinone Alkaloid and Polyketide from the *Cordyceps*-Colonizing Fungus *Pseudogymnoascus roseus*

**DOI:** 10.3390/biom16020187

**Published:** 2026-01-26

**Authors:** Jie Lin, Yutong Guo, Jing Wang, Fang Wang, Ling Liu

**Affiliations:** 1 Jiangsu Key Laboratory for Biofunctional Molecules, College of Life Science and Chemistry, Jiangsu Second Normal University, Nanjing 210013, China; linjie@jssnu.edu.cn; 2 State Key Laboratory of Microbial Diversity and Innovative Utilization, Institute of Microbiology, Chinese Academy of Sciences, University of Chinese Academy of Sciences, Beijing 100101, China; 13770309523@163.com (Y.G.); 202401932015@sxmu.edu.cn (J.W.); 3 Shandong Academy of Pharmaceutical Sciences, Jinan 250101, China; wangfang-zy@sdaps.cn

**Keywords:** fungus, *Cordyceps*-colonizing, fungus, structure, elucidation, cytotoxicity, network pharmacology, molecular docking

## Abstract

One new pyridinone alkaloid pseudogymnone A (**1**) and one new tricyclic polyketide penijanthinone C (**2**), together with six known compounds, harzianic acid (**3**), 3-methyl-2-(2-nonenyl)-4(1*H*)-quinolinone (**4**), emodic acid (**5**), alaternin (**6**), violaceol-I (**7**), and violaceol-II (**8**), were obtained from the *Cordyceps*-colonizing fungus *Pseudogymnoascus roseus*. The structures and absolute configurations of the isolated compounds were elucidated through a combination of NMR and MS spectroscopic analyses, ECD calculations, and X-ray crystallography. Compound **3** exhibited obvious cytotoxicity against A549 (IC_50_ = 4.2 µM) and MGC (IC_50_ = 3.8 µM) cell lines. Integrated network pharmacology and molecular docking analyses indicated that compound **3** exerts potential anti-gastric-cancer effects by modulating multiple cancer-related signaling pathways, with EGFR identified as a potential target of compound **3**.

## 1. Introduction

Natural fungal products are pharmacologically promising and structurally diverse, serving as crucial sources for drug discovery [1]. In 2023 and 2024 alone, more than 1400 new natural fungal products were reported, including polyketides, terpenoids, alkaloids, peptides, and steroids [2,3]. These metabolites display a broad spectrum of bioactivities including anti-inflammatory, antifungal, antibacterial, cytotoxic, and enzyme inhibitory effects, with some outperforming clinical drugs [2,3]. Fungi thriving in specialized and competitive environments tend to biosynthesize diverse structural secondary metabolites [4], which are not only crucial for their survival and growth but also exhibit various biological activities with potential pharmaceutical applications [5]. Recent studies on secondary metabolites produced by *Cordyceps*-colonizing fungi, inhabiting the fruiting bodies of *Cordyceps sinensis*, have revealed novel structural compounds with promising bioactivities. Examples include a novel gentianellane-type sesterterpenoid versicolorin A, which showed significant *β*-glucuronidase inhibitory activity and was isolated from *Aspergillus versicolour* ZJUTE2 [6]; the antifungal 4*H*-chromen-4-one derivatives coniochaetones E–I, obtained from *Fimetariella* sp. [7]; the cytotoxic *p*-terphenyls gliocladinin A and its *β*-d-glucoside gliocladinin B, isolated from *Gliocladium* sp. strain XZC04-CC-302 [8]; and the cytotixic cycloaspeptides F and G, derived from *Isaria farinose* [9]. Therefore, *Cordyceps*-colonizing fungi are prolific sources of bioactive natural fungal products with promising potential for future pharmacological development.

**Figure 1:**
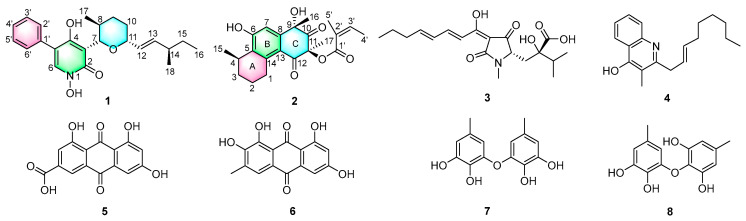
Chemical structures of compounds **1**–**8**.

Our research aims to discover bioactive metabolites produced by fungi residing in underexplored ecological niches. We obtained the fungal strain *Pseudogymnoascus roseus* by isolating it from the sclerotia of *Cordyceps sinensis* (Berk.) Sacc., which were collected from the cold, alpine environment of Kangding, Sichuan Province, China. A subsequent chemical analysis of the crude extract of *P. roseus* produced one new pyridinone alkaloid pseudogymnone A (**1**) and one new tricyclic polyketide penijanthinone C (**2**) together with six known compounds: harzianic acid (**3**) [10], 3-methyl-2-(2-nonenyl)-4(1*H*)-quinolinone (**4**) [11], emodic acid (**5**) [12], alaternin (**6**) [13], violaceol-I (**7**) [14], and violaceol-II (**8**) [14] (Figure 1). Cytotoxicity assays revealed that compound **3** exhibited significant inhibitory effects against gastric cancer cell lines. A network pharmacology analysis was applied to predict the possible mechanism of compound **3**, which was further validated by molecular docking. The comprehensive details of the isolation, structural characterization, biological evaluation, network pharmacology analysis, and molecular docking of these compounds are described herein.

## 2. Materials and Methods

### 2.1. Molecular Identification

To identify the isolate, we combined a morphological examination with a molecular analysis of the rDNA ITS region (GenBank Accession No. DQ117451). This fungus was deposited at the Institute of Microbiology, Chinese Academy of Sciences, Beijing, under the accession number S161.

### 2.2. General Experimental Procedure

The experimental approaches employed in this study were adapted from previously reported protocols [15]. Comprehensive descriptions of the methods are included in the Supplementary Method S1.

### 2.3. Fungal Materials, Cultivation, Fermentation, and Isolation

The fungus *P. roseus* was isolated from the sclerotia of *C. sinensis* (Berk.) Sacc. collected from Kangding, Sichuan, China. After 10 days of incubation on PDA at 25 °C, mycelial agar plugs (0.5 cm^3^ each) were used to inoculate a liquid seed medium (1% malt extract, 0.4% glucose, and 0.4% yeast extract, pH 6.5) in an Erlenmeyer flask. Shake cultivation (170 rpm, 5 d, 25 °C) provided the seed culture. For large-scale fermentation, autoclaved rice in Fernbach flasks was inoculated with a spore suspension (1 × 10^6^ spores/mL) and incubated statically at 25 °C for 40 days.

The fermentation product was extracted multiple times with ethyl acetate (4 × 500 mL). The combined organic layers were concentrated under reduced pressure to afford a crude extract (4.2 g). This crude material was subjected to vacuum column chromatography over silica gel, eluted with a gradient of petroleum ether–ethyl acetate. The 30% ethyl acetate fraction (286 mg) was collected and further purified by Sephadex LH-20 gel column chromatography (Cytiva, Uppsala, Sweden; eluent: 1:1 CH_2_Cl_2_–MeOH). The combined relevant fractions were then refined by reversed phase HPLC (column: Agilent Zorbax SB-C18, 9.4 × 250 mm, 5 µm; elution program: 20% MeOH–H_2_O isocratic for 2 min, followed by a gradient from 20% to 90% MeOH–H_2_O over 50 min; flow rate: 2 mL/min, Agilent Technologies, Santa Clara, CA, USA) to yield compound **1** (4.7 mg, *t*_R_ 48.9 min) and compound **2** (7.2 mg, *t*_R_ 39.5 min).The 60% ethyl acetate fraction (123 mg) was purified by RP-HPLC (20% MeOH–H_2_O for 5 min, followed by a gradient from 20% to 80% MeOH–H_2_O over 30 min), affording compound **3** (6.9 mg, *t*_R_ 27.6 min). Similarly, the 70% ethyl acetate fraction (261 mg) was separated by RP-HPLC (25% MeOH–H_2_O for 5 min, followed by a gradient from 25% to 80% MeOH–H_2_O over 30 min), yielding compounds **4** (6.2 mg, *t*_R_ 23.2 min), **5** (7.4 mg, *t*_R_ 17.6 min), **6** (4.1 mg *t*_R_ 15.4 min), **7** (5.1 mg, *t*_R_ 26.3 min), and **8** (3.7 mg, *t*_R_ 25.4 min).

Pseudogymnone A (**1**): yellow oil; ${\left[\alpha \right]}_{D}^{25}$ = +252 (*c* 0.3, CH_3_OH); UV (MeOH) *λ*_max_ (log *ε*) 207.1 (2.13), 241.0 (1.92), 296.0 (0.88) nm; IR (neat) *ν*_max_ 3192, 2962, 2930, 1641, 1556, 1455, 1225 cm^−1^. ^1^H, ^13^C NMR and HMBC data see Table 1 and Supplementary Figures S1–S3; HR-ESIMS *m*/*z* 406.1989 [M + Na]^+^ (calcd for C_23_H_29_NO_4_Na, 406.1994).

Penijanthinone C (**2**): yellow powder; ${\left[\alpha \right]}_{D}^{25}$ = +71 (*c* 0.1, CH_3_OH); UV (MeOH) *λ*_max_ (log *ε*) 218.0 (2.46), 229.0 (2.58), 288 (1.76) nm; IR (neat) *ν*_max_ 3192, 2962, 2930, 1641, 1556, 1455, 1225 cm^−1^. ^1^H, ^13^C NMR, and HMBC data see Table 2 and Supplementary Figures S7, S8 and S11; HR-ESIMS *m*/*z* 385.1650 [M − H]^−^ (calcd for C_22_H_25_O_6_, 385.1651).

### 2.4. X-Ray Crystal Structure Analysis

A single crystal of compound **2** was obtained by employing the vapor-phase diffusion technique utilizing acetone−H_2_O (10:1), and data were collected using a Rigaku RAPID IP diffractometer (Rigaku, Tokyo, Japan) with graphite-monochromated Mo K*α* radiation, *λ* = 0.71073 Å at 173(2) K. Crystal data: C_22_H_26_O_6_, 2(CH_4_O), *M* = 450.51, space group orthorhombic, *P*2(1)2(1)2(1); unit cell dimensions *a* = 7.2615(15) Å, *b* = 10.299(2) Å, *c* = 31.986(6) Å, *V* = 2392.2(8) Å^3^, *Z* = 4, *D*_(calcd)_ = 1.251 mg/mm^3^, *μ* = 0.093 mm^−1^, *F*(000) = 968. Crystallographic data for compound **2** have been deposited with the Cambridge Crystallographic Data Centre (deposition number CCDC 2518640). The crystal structure was solved and refined using SHELXL-97 [16] with anisotropic non-H atoms and riding H atoms. After SADABS absorption correction, 5487 unique reflections produced a final *R*_1_ = 0.0456 and *wR*_2_ = 0.1085 [*I* > 2*σ*(*I*)].

### 2.5. ECD Calculation Methods

The absolute configurations of compounds **1** and **2** were assigned by comparing their experimental ECD spectra with the Boltzmann-weighted TD-DFT simulations. Full details regarding the computational procedures are provided in the Supplementary Method S2.

### 2.6. MTS Assay

Cell proliferation was evaluated with the MTS assay, following established protocols, and IC_50_ values were determined via nonlinear regression (SPSS Statistics 19.0) [17,18]. The human cancer cell lines used in this assay have been described in our previous work [19]. Further experimental details are available in the Supplementary Method S3.

### 2.7. Network Pharmacology

The SMILES of compound **3** was submitted to SwissTargetPrediction (http://www.swisstargetprediction.ch/ (accessed on 11 November 2025)), PharmMapper (http://lilab-ecust.cn/pharmmapper/ (accessed on 11 November 2025)), and TargetNet (http://targetnet.scbdd.com/ (accessed on 11 November 2025)) to predict its potential targets. Gastric cancer-related targets were collected from the GeneCards (https://www.genecards.org/ (accessed on 11 November 2025)) and OMIM (https://www.omim.org/ (accessed on 11 November 2025)) databases.

A Venn diagram of common targets between compound **3** and gastric cancer was constructed using Venny 2.1.0. Subsequently, the overlapping targets were processed through the STRING (https://string-db.org/ (accessed on 20 November 2025)) database to construct a protein–protein interaction (PPI) network. PPIs with a medium confidence (score > 0.4) were included. Cytoscape 3.9.1 was used to construct the key target network and to systematically analyze network parameters. To elucidate the potential mechanism of compound **3** against gastric cancer, we performed a functional enrichment analysis using the Database for Annotation, Visualization, and Integrated Discovery (DAVID) (https://davidbioinformatics.nih.gov/ortholog.jsp (accessed on 2 December 2025)).

### 2.8. Molecular Docking

The crystal structure of the human EGFR ligand-binding domain (PDB ID: 9GFE) was retrieved from the Protein Data Bank. The 3D structure of harzianic acid (PubChem CID: 139585999) was obtained from PubChem and energy minimized under the MMFF94 force field. Molecular docking simulations were performed with AutoDock Vina 1.2.3 The receptor was prepared with PyMOL 2.5.5 by removing crystallographic water molecules, ions, and any co-bound ligands. A docking grid that enclosed the entire protein was defined using AutoDock Vina 1.2.3. Both the receptor and ligand were converted to the required PDBQT format with ADFRsuite 1.0^3^. The exhaustiveness parameter for the global search was set to 32; all other parameters were kept at their default values. The top-ranked pose reported by Vina was taken as the binding mode and subsequently visualized with PyMOL 2.5.5.

### 2.9. Use of AI-Assisted Writing Tools

During the preparation of this manuscript, Kimi was employed as an auxiliary tool for grammar checking and sentence structure refinement to enhance the readability of the text. It is crucial to emphasize that all critical academic arguments, data interpretations, and conclusions originated solely from and are the full responsibility of the authors. The tools were strictly confined to optimizing the clarity of the language.

## 3. Results

### 3.1. Structure Elucidation

**Figure 2:**
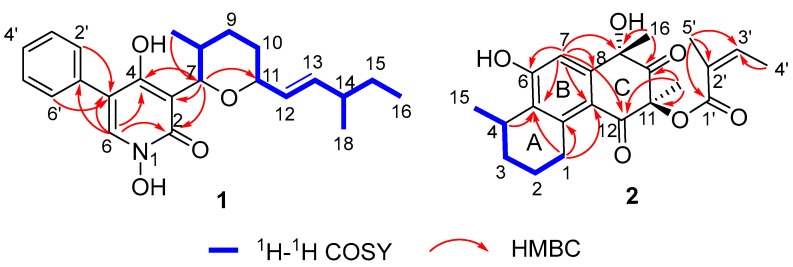
Key ^1^H-^1^H COSY and HMBC correlations of compounds **1** and **2**.

The molecular formula of pseudogymnone A (**1**) was assigned as C_23_H_29_NO_4_Na based on its HRESIMS (*m*/*z* 406.1989 [M + Na]^+^; Δ –0.5 mmu). The analysis of its ^1^H and ^13^C NMR data (Table 1) and HSQC spectrum (Figure S4) showed the presence of one carbonyl amide carbon (*δ*_C_ 157.4), twelve aromatic or olefinic carbons (including eight sp^2^ methines and four sp^2^ quaternary carbon), four sp^3^ methines (two oxygenated), three methylenes, three methyl groups, and one hydroxyl proton (*δ*_H_ 9.63). The NMR signals of the aromatic region of compound **1** matched well with those of the 1,4-dihydroxy-5-phenyl-2-pyridinone moiety, which is a key skeleton of the anticancer agent TMC-69 [14]. HMBC correlations from H-6 to C-2, C-4, C-5, and C-1′, and from H-2′ and H-6′ to C-5, together with ^1^H-^1^H COSY correlations (Figure 2 and Figure S5), as well as IR absorptions (3192, 1641, and 1556 cm^−1^), further confirmed the presence of 1,4-dihydroxy-5-phenyl-2-pyridinone moiety. ^1^H-^1^H COSY correlations (Figure 2) define the isolated spin system of the side chain from the C-7 to C-17 subunit. In addition, HMBC correlations from H-7 (*δ*_H_ 4.85) to C-11 (*δ*_C_ 79.8) and from H-11 (*δ*_H_ 4.00) to C-7 (*δ*_C_ 80.9) indicated that C-7 and C-11 were connected via the same oxygen atom to form the tetrahydropyran ring. Key HMBC correlations from H-7 to C-4, C-3, and C-2 identified the tetrahydropyran ring at the C-3 position of the pyridinone moiety, completing the planar structure of compound **1,** as described in Figure 1.

The relative configuration of pseudogymnone A (**1**) was established based on NOESY correlations and the ^1^H NMR coupling constants. The *trans*-diaxial orientation of H-8 and H-7 was determined by the large ^3^*J*_7,8_ value of 8.5 Hz. The C-12/C-13 olefinic bond was determined as *E*-geometry based on the large coupling constant (14 Hz) of H-12 and H-13. Additionally, NOESY correlations (Supplementary Figure S6) observed for H-11 with H-7 and H_3_-17 revealed that H-11, H-7, and H_3_-17 are on the same face of the tetrahydropyran ring. However, the configuration of C-14 remained unassigned owing to the lack of relevant NOESY cross-peaks.

**Figure 3:**
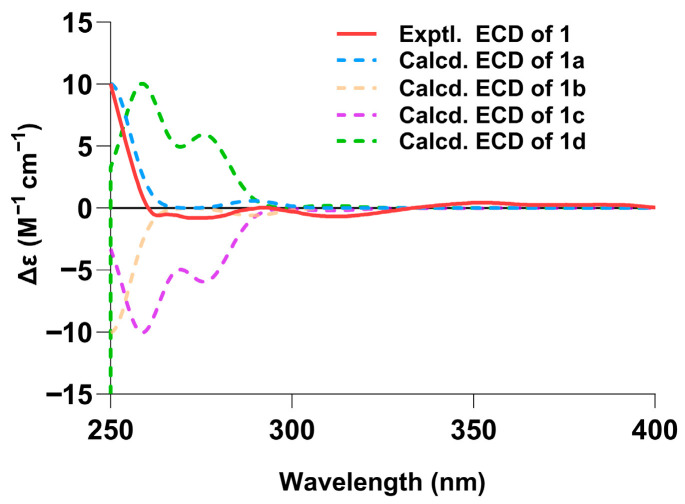
Experimental ECD of compound **1** in MeOH and the calculated ECD spectra of **1a**–**1d**.

ECD calculations at the CAM-B3LYP/6-311G (d,p) level were performed for four stereoisomers: (7*S*,8*S*,11*R*,14*R*)-**1a,** (7*S*,8*S*,11*R*,14*S*)-**1b,** (7*R*,8*R*,11*S*,14*S*)-**1c,** and (7*R*,8*R*,11*S*,14*R*)-**1d**. The subsequent comparison revealed that the experimental ECD curve of compound **1** (Figure 3 and Table S1) aligned conclusively with the Boltzmann-weighted theoretical spectrum of (7*S*,8*S*,11*R*,14*R*)-**1a**, thereby establishing its absolute configuration (Figure 3).

Penijanthinone C (**2**) was obtained as a pale-yellow powder. Its molecular formula was assigned as C_22_H_26_O_6_ (corresponding to 10 degrees of unsaturation) based on HRESIMS (*m*/*z* 385.1650 [M − H]^−^; Δ –0.1 mmu). The 1D NMR data of compound **2** (Table 2) and HSQC spectrum (Figure S9) displayed signals for two exchangeable protons (*δ*_H_ 5.80 and 3.43), five methyl groups, three methylenes, one sp^3^ methine, eight olefinic or aromatic carbons (two protonated), two sp^3^ quaternary carbons that are oxygenated (*δ*_C_ 76.2 and 83.4, respectively), one carbonyl carbon (*δ*_C_ 206.7), one *α*,*β*-unsaturated carbonyl carbon (*δ*_C_ 191.9), and one unsaturated ester carbonyl carbon (*δ*_C_ 166.1). These data were consistent with all NMR resonances and implied that compound **2** contains three rings. The comparison of the NMR data for compound **2** with those of penijanthinone A suggested a similar tricyclic polyketide core [20]. HMBC correlations (Figure 2 and Figure S11) from H_2_-1 to C-5, C-13, and C-14; from H-4 to C-5; and from H-7 to C-5, C-6, C-8, and C-13 as well as ^1^H-^1^H COSY correlations (Figure 2 and Figure S10) of H_2_-1/H_2_-2/H_2_-3/H-4/H_3_-15 established a tetrahydronaphthalenol substructure (rings A and B), with the C-15 methyl group attached to the C-4 position. Further HMBC cross-peaks from H_3_-16 to C-8, C-9, and the carbonyl carbon C-10 (*δ*_C_ 206.7); from H_3_-17 to C-11, C-10, and C-12 (*δ*_C_ 191.9); and from H-7 to C-9 and C-12 indicated the presence of a cyclohexane-1,3-dione moiety (ring C) fused to the tetrahydronaphthalenol substructure at C-8 and C-13, thus completing the tetrahydrophenanthrene-2,4(1*H*,3*H*)-dione tricyclic core skeleton. Other HMBC correlations from H-3′ to the C-1′ (*δ*_C_ 166.1) ester carbonyl carbon; C-5′, C-4′, and C-2′ from H_3_-4′ to C-3′ and C-2′; and from H_3_-5 to C-3′, C-2′, and C-1′ established a 2-methylbut-2-enoic acid moiety located at the C-11 position of tetrahydrophenanthrene-2,4(1*H*,3*H*)-dione to form 2-methylbut-2-enoate. This was further confirmed based on the chemical shift in the oxygenated sp^3^ quaternary carbon C-11 (*δ*_C_ 83.4) and the molecular weight of compound **2**.

**Figure 4:**
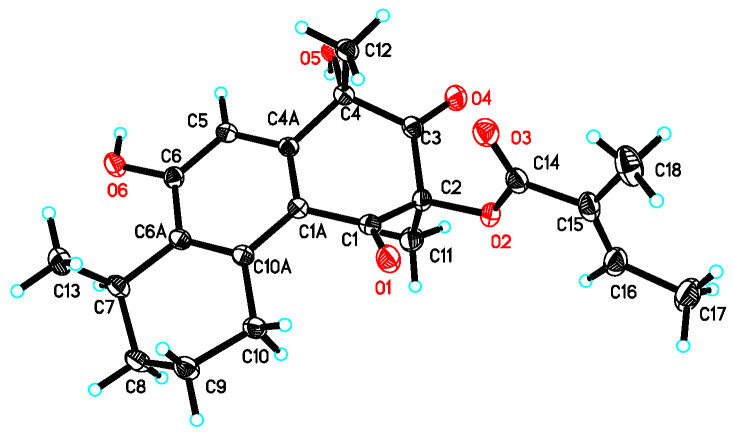
X-ray crystallography ORTEP plot of compound **2**.

Ultimately, the structure of compound **2** was unequivocally confirmed by the single-crystal X-ray crystallography (ORTEP plot), which also enabled the determination of its relative configuration, as shown in Figure 4.

**Figure 5:**
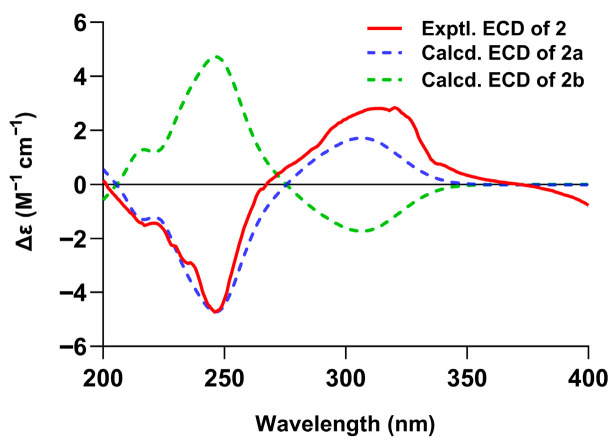
Experimental ECD of compound **2** in MeOH and calculated ECD spectra for **2a** and **2b**.

The absolute configuration of compound **2** was determined by comparing its ECD spectrum with those calculated via the TDDFT method at the CAM-B3LYP/6-311G(d,p) level for two possible stereoisomers (4*R*,9*S*,11*S*)-**2a** and (4*S*,9*R*,11*R*)-**2b** (Figure 5 and Table S2). The close match with the calculated spectrum of compound **2a** confirmed the absolute configuration of 4*R*,9*S*,11*S* for compound **2**.

The known compounds **3**–**8**, isolated from the crude extract, were identified as harzianic acid (**3**) [10], 3-methyl-2-(2-nonenyl)-4(1*H*)-quinolinone (**4**) [11], emodic acid (**5**) [12], alaternin (**6**) [13], violaceol-I (**7**) [14], and violaceol-II (**8**) [14], respectively, based on comparative NMR and MS analyses with published data.

### 3.2. Cytotoxicity

Compounds **1**–**3** were tested for cytotoxicity against a panel of human tumor cell lines. Compound **3** exhibited significant activity, with IC_50_ values of 3.8 µM (against MGC cells) and 4.2 µM (against A549 cells). In contrast, the positive control paclitaxel exhibited potent activity against the tested cells, showing low IC_50_ values (e.g., 1.6 × 10^−5^ µM for Hep3B-2 cells) (Table 3).

### 3.3. Network Pharmacology Prediction for Compound **3**

**Figure 6:**
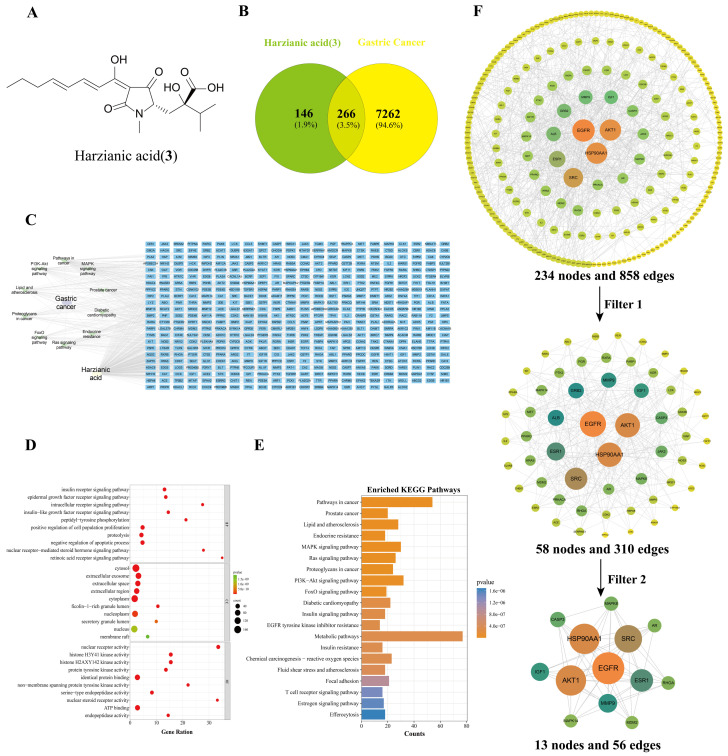
Identification and analysis of intersection targets shared by harzianic acid (**3**) and gastric cancer. (**A**) Chemical structure of harzianic acid (**3**). (**B**) Venn diagram showing the overlapping targets harzianic acid (**3**) and gastric cancer. (**C**) Compound–disease–target network of harzianic acid (**3**) and gastric cancer. (**D**,**E**) Go functional enrichment analysis and KEGG pathway analysis of harzianic acid (**3**) and gastric cancer. (**F**) The PPI network of harzianic acid (**3**) and gastric cancer.

The intersection of compound **3** and gastric cancer was visualized by a Venn diagram, identifying 266 intersection targets (Figure 6B). Subsequently, a “compound–gastric cancer target” network (Figure 6C) was established by Cytoscape 3.9.1.

To delineate the mechanism, GO functional annotation and KEGG pathway enrichment analyses were performed on the shared targets. The GO enrichment analysis (Figure 6D) revealed that these targets were primarily involved in receptor-mediated signaling transduction, peptidyl-tyrosine phosphorylation, and the regulation of cell proliferation and apoptosis. Furthermore, the molecular functions of these targets were varied, ranging from nuclear receptor and protein tyrosine kinase activity to serine-type endopeptidase and histone kinase activity. These molecular functions are closely associated with the processes of abnormal proliferation, apoptosis evasion, and invasion–metastasis in gastric cancer cells [21,22]. Importantly, the KEGG pathway enrichment analysis (Figure 6E) indicates that the major enriched pathways included the cancer pathway MAPK and PI3K-Akt signaling pathways.

These intersection targets were imported into STRING to construct a PPI network, which was then topologically analyzed through two rounds of CytoNCA screening using median value cutoffs (Figure 6F). This analysis identified 13 core targets, including EGFR, HSP90AA1, AKT1, and others. They are involved in several classical signaling pathways, including PI3K-Akt and MAPK signaling pathways, as well as pathways in cancer [23], which aligned with the findings from the KEGG pathway enrichment analysis. As a receptor tyrosine kinase, the key gene EGFR regulated multiple signaling pathways associated with cell proliferation, apoptosis, invasion, and metastasis [24]. Collectively, these synergistic multi-target interactions may cooperatively modulate the gastric cancer microenvironment and contribute to the reversal of the malignant phenotype.

### 3.4. Molecular Docking Analysis of Compound **3**

**Figure 7:**
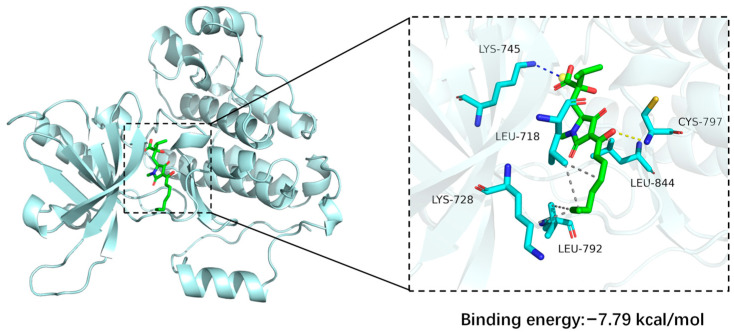
The molecular docking of harzianic acid (**3**) complexed with the EGFR. In the figure, the small molecule is shown as green sticks, and the protein is represented by a light-cyan cartoon. Hydrogen bond, hydrophobic, and salt bridge interactions are shown as yellow, gray, and blue dashed lines, respectively.

The interaction analysis between the EGFR and compound **3** indicates that the protein and ligand form a stable complex predominantly driven by electrostatic interactions, together with hydrophobic interactions and hydrogen bonds (Figure 7). Notably, a salt bridge interaction is formed between the ligand and the EGFR: the side chain ammonium group of Lys745 serves as a positive charge center and establishes a salt bridge with the carboxyl group of compound **3** [25]. With respect to hydrophobic interactions, multiple hydrophobic residues form a compact hydrophobic network with the ligand [26]. Specifically, Leu718 establishes dual hydrophobic contacts with the carbon skeleton of the ligand, and Lys728, Leu792, and Leu844 also participate in hydrophobic interactions, while the main chain amino group of Cys797 interacts with the oxygen atom of the ligand to form a hydrogen bond. Although the number of hydrogen bonds is relatively limited, this interaction acts synergistically with the salt bridge and hydrophobic interactions to stabilize the complex. As a result, the ligand achieves stable and specific binding by forming a salt bridge with Lys745 within the protein binding pocket, engaging in dense hydrophobic interactions with multiple leucine residues, and being further stabilized by a hydrogen bond involving Cys797. This binding mode, characterized by electrostatic interactions as the dominant driving force in concert with multiple non-covalent interactions, provides an important structural basis for elucidating the molecular recognition mechanism between the ligand and protein. A negative binding affinity indicates the feasibility of binding, and, generally, a lower value corresponds to stronger binding [27]. In this complex, the docking results yielded a binding affinity of −7.79 kcal/mol for the EGFR and harzianic acid (**3**), indicating a favorable binding interaction between the protein and the ligand.

## 4. Discussion

Pseudogymnone A (**1**) is a new pyridinone alkaloid that shares the same core skeleton as the known compound TMC-69 [28] but differs in its substituents. Specifically, compound **1** features a 3-methyl-1-pentenyl group at C-11, whereas TMC-69 lacks substitution at C-11 and instead possesses a longer 6-methyl-1,3,5-octatrienyl polyene chain at the C-10 position, while penijanthinone C (**2**) is a new tricyclic polyketide, bearing a similar structural skeleton to penijanthinones A and B [20]. The main difference is that compound **2** is substituted by a side chain at the C-11 position, whereas penijanthinone A carries a longer side chain at the C-3 position. Furthermore, compound **2** lacks two hydroxyl groups located at C-2 and C-4 of penijanthinone A.

In addition, bioactivity assays showed that harzianic acid (**3**) exhibited selective cytotoxicity against both MGC (IC_50_ = 3.8 µM) and A549 (IC_50_ = 4.2 µM) cell lines. Harzianic acid (**3**) was first described in 1994 as an antifungal agent [10]. Compound **3** inhibited the growth of *Pythium irregulare* and *Sclerotinia sclerotiorum* [29], eradicated methicillin-resistant *Staphylococcus pseudintermedius* at 16 mg/L [30], and acted as a Gram-positive-selective membrane disruptor of *Bacillus subtilis* [31]. Furthermore, compound **3** has also been identified as a novel natural product inhibitor of fungal acetohydroxyacid synthase (AHAS), retaining efficacy against herbicide-resistant enzyme variants. Its selectivity, arising from a unique binding mode involving the C3′ side chain that is incompatible with the plant AHAS active site, coincides with its reported plant growth-promoting properties [32]. To date, only one brief report has noted that compound **3** exhibits weak anti-liver-cancer activity with an IC_50_ of 58.92 ± 1.81 μM against SMMC-7721 cells.

The molecular docking analysis predicted that compound **3** binds to the EGFR with a binding affinity of −7.79 kcal mol^−1^, with the carboxyl group of compound **3** forming a salt bridge to Lys745; hydrophobic interactions with Leu718, Leu792, and Leu844; and a hydrogen bond with Cys797, consistent with the network pharmacology prediction that the EGFR is a potential key target of this compound. This finding aligns with the ongoing evolution of EGFR-directed anticancer agents, which have progressed from conventional monoclonal antibodies [33] to innovative fusion proteins [34] and naturally derived small molecules [35]. However, the present work is constrained by the low fermentation yield of harzianic acid (**3**), which limited further bioactivity evaluations beyond cytotoxicity assays. Moreover, its proposed interaction with the EGFR is based solely on network pharmacology and molecular docking predictions.

## 5. Conclusions

In summary, two new compounds, pseudogymnone A (**1**, a pyridinone alkaloid) and penijanthinone C (**2**, a tricyclic polyketide), along with six known compounds were obtained from the fungus *P. roseus*. The biological evaluation revealed that harzianic acid (**3**) exhibits potent and selective cytotoxicity against gastric cancer (MGC) and lung adenocarcinoma (A549) cell lines. Integrated network pharmacology predictions and molecular docking analyses implied that the EGFR is a potential target of compound **3**. This study expands the chemical space of *Cordyceps*-colonizing fungal metabolites and identifies compound **3** as a potential EGFR-interacting scaffold that merits further experimental validation as part of future anti-gastric-cancer drug discovery efforts.

## Figures and Tables

**Table 1:** NMR data of pseudogymnone A (**1**) in CDCl_3_.

Pos.	*δ*_C_, Mult.	*δ*_H_ *J* (Hz)	HMBC
2	157.4, C		
3	109.8, C		
4	161.2, C		
5	114.2, C		
6	131.1, CH	7.68, s	1′, 2, 4, 5
7	80.9, CH	4.85, d (10.3)	2, 3, 4, 10, 11
8	35.8, CH	1.89, m	7, 8, 11
9a	32.4, CH_2_	1.93, m	7, 8, 11
9b		1.48, m	10, 11
10a	32.5, CH_2_	1.79, d	8
10b		1.48, m	9, 11
11	79.8, CH	4.00, m	7, 9, 12, 13
12	127.9, CH	5.39, dd (15.6, 6.0)	10, 11, 13, 14
13	138.8, CH	5.57, dd (15.6, 7.3)	11, 12, 14, 15, 18
14	38.0, CH	2.0, m	12, 13, 15, 16, 18
15	29.5, CH_2_	1.28, m	13, 14, 16, 18
16	11.7, CH_3_	0.83, d (3.3)	14, 15
17	17.6, CH_3_	0.84, d (2.1)	8, 9, 10
18	19.6, CH_3_	0.94, d (6.7)	13, 14, 15
1′	133.3, C		
2′	129.3, CH	7.47, d (7.3)	4′, 5, 6′
3′	128.6, CH	7.42, t (7.5)	1′, 5′
4′	127.9, CH	7.35, t (7.2)	2′
5′	128.6, CH	7.42, t (7.5)	1′, 3′
6′	129.3, CH	7.47, d (7.3)	2′, 4′, 5
OH		9.63, br s	

**Table 2:** NMR data of penijanthinone C (**2**) in CDCl_3_.

Pos.	*δ*_C_, Mult.	*δ*_H_ *J* (Hz)	HMBC
1a	27.2, CH_2_	3.68, dt (4.7, 18.7)	2, 3, 14
1b		2.51, ddd (6.1, 9.4, 18.2)	2, 3, 5, 13, 14
2a	18.2, CH_2_	1.77, m	4
2b		1.69, m	4, 14
3a	29.3, CH_2_	1.77, m	1, 2, 4, 15
3b		1.66, m	1, 4, 15
4	27.2, CH	3.15, dt (4.7, 9.5)	5, 15
5	130.2, C		
6	158.2, C		
7	109.4, CH	7.07, s	5, 6, 8, 9, 12, 13
8	143.6, C		
9	76.2, C		
10	206.7, C		
11	83.4, C		
12	191.9, C		
13	120.6, C		
14	142.2, C		
15	20.6, CH_3_	1.27, d (6.8)	3, 4, 5
16	35.4, CH_3_	1.73, s	8, 9, 10
17	23.4, CH_3_	1.49, s	10, 11, 12
1′	166.1, C		
2′	127.2, C		
3′	139.8, CH	7.08, m	1′, 2′, 4′, 5′,
4′	14.6, CH_3_	1.84, d (7.1)	2′, 3′
5′	12.0, CH_3_	1.88, s	1′, 2′, 3′
-OH		5.80, s	
-OH		3.43, s	

**Table 3:** Cytotoxicity of compounds **1**–**3**.

IC_50_ (μM)				
Compounds	1	2	3	Paclitaxel
A549	>100	>100	4.2 ± 0.1	(3.0 ± 0.2) × 10^−2^
CNE1-LMP1	>100	>100	>100	(4.2 ± 0.1) × 10^−3^
A375	>100	>100	82.3 ± 1.0	(8.9 ± 0.2) × 10^−3^
MCF-7	>100	>100	65.7 ± 1.8	(1.4 ± 0.1) × 10^−2^
MGC	>100	>100	3.8 ± 0.3	(6.4 ± 0.2) × 10^−3^
EC109	>100	>100	>100	(1.4 ± 0.1) × 10^−2^
PANC-1	>100	>100	>100	(1.1 ± 0.06) × 10^−3^
Hep3B-2	>100	>100	>100	(1.6 ± 0.04) × 10^−5^
HaCaT	>100	>100	>100	(2.4 ± 0.2) × 10^−2^

## Data Availability

All data generated or analyzed in this study are available within the manuscript and are available from the corresponding author upon request.

## Supplementary Materials

The following Supplementary Materials can be downloaded at https://www.mdpi.com/article/10.3390/biom16020187/s1: Method S1. General experimental procedure; Method S2. ECD calculation methods; Method S3. MTS assay; Figure S1. ^1^H NMR spectrum of pseudogymnone A (**1**; 500 MHz, CDCl_3_); Figure S2. ^13^C NMR spectrum of pseudogymnone A (**1**; 125 MHz, CDCl_3_); Figure S3. HMBC spectrum of pseudogymnone A (**1**; 500 MHz, CDCl_3_); Figure S4. HSQC spectrum of pseudogymnone A (**1**; 500 MHz, CDCl_3_); Figure S5. ^1^H-^1^H COSY spectrum of pseudogymnone A (**1**; 400 MHz, CDCl_3_); Figure S6. NOESY spectrum of pseudogymnone A (**1**; 500 MHz, CDCl_3_); Figure S7. ^1^H NMR spectrum of penijanthinone C (**2**; 600 MHz, CDCl_3_). Figure S8. ^13^C NMR spectrum of penijanthinone C (**2**; 125 MHz, CDCl_3_); Figure S9. HSQC spectrum of penijanthinone C (**2**; 600 MHz, CDCl_3_); Figure S10. ^1^H-^1^H COSY spectrum of penijanthinone C (**2**; 600 MHz, CDCl_3_); Figure S11. HMBC spectrum of penijanthinone C (**2**; 600 MHz, CDCl_3_); Table S1. ECD conformers of pseudogymnone A (**1**); and Table S2. ECD conformers of penijanthinone C (**2**).

## Abbreviations

NMR	Nuclear Magnetic Resonance
MS	Mass Spectrometry
ECD	Electronic Circular Dichroism
IR	Infrared
ESIMS	Electrospray Ionization Mass Spectrometry
HRESIMS	High-Resolution Electrospray Ionization Mass Spectrometry
CC	Column Chromatography
RP HPLC	Reversed Phase High-Performance Liquid Chromatography
MeOH	Methanol
CH_2_Cl_2_	Dichloromethane
EtOAc	Ethyl Acetate
PDA	Potato Dextrose Agar
PPIs	Protein–Protein Interactions
GO	Gene Ontology
KEGG	Kyoto Encyclopedia of Genes and Genomes
DAVID	Database for Annotation, Visualization, and Integrated Discovery
PDB	Protein Data Bank
TDDFT	Time-Dependent Density Functional Theory
COSY	Correlation Spectroscopy
HMBC	Heteronuclear Multiple-Bond Correlation
HSQC	Heteronuclear Single Quantum Coherence
NOESY	Nuclear Overhauser Effect Spectroscopy
DMSO	Dimethyl Sulfoxide
EGFR	Epidermal Growth Factor Receptor
