# Supplementary material for: New Pyridinone Alkaloid and Polyketide from the Cordyceps-Colonizing Fungus Pseudogymnoascus roseus
Source: Biomolecules. 2026 Jan 26;16(2):187. doi: 10.3390/biom16020187 (PMC12938783; doi:10.3390/biom16020187)
Supplement: Supplementary file 1 [file biomolecules-16-00187-s001.zip › biomolecules-4102742-supplementary.pdf]

Supplementary Materials

New Pyridinone Alkaloid and Polyketide from the *Cordyceps*-Colonizing Fungus *Pseudogymnoascus roseus*

Jie Lin [1], Yutong Guo [2], Jing Wang [2], Fang Wang [3] and Ling Liu [2,*]

[1] Jiangsu Key Laboratory for Biofunctional Molecules, College of Life Science and Chemistry, Jiangsu Second Normal University, Nanjing 210013, China; linjie@jssnu.edu.cn
[2] State Key Laboratory of Microbial Diversity and Innovative Utilization, Institute of Microbiology, Chinese Academy of Sciences, University of Chinese Academy of Sciences, Beijing 100101, China; 13770309523@163.com (Y.G.); 202401932015@sxmu.edu.cn (J.W.)
[3] Shandong Academy of Pharmaceutical Sciences, Jinan 250101, China; wangfang-zy@sdaps.cn
* Correspondence: liul@im.ac.cn

Abstract

One new pyridinone alkaloid pseudogymnone A (**1**) and one new tricyclic polyketide penijanthinone C (**2**), together with six known compounds, harzianic acid (**3**), 3-methyl-2-(2-nonenyl)-4(1*H*)-quinolinone (**4**), emodic acid (**5**), alaternin (**6**), violaceol-I (**7**), and violaceol-II (**8**), were obtained from the *Cordyceps*-colonizing fungus *Pseudogymnoascus roseus*. The structures and absolute configurations of the isolated compounds were elucidated through a combination of NMR and MS spectroscopic analyses, ECD calculations, and X-ray crystallography. Compound **3** exhibited obvious cytotoxicity against A549 (IC_{50} = 4.2 μM) and MGC (IC_{50} = 3.8 μM) cell lines. Integrated network pharmacology and molecular docking analyses indicated that compound **3** exerts potential anti-gastric-cancer effects by modulating multiple cancer-related signaling pathways, with EGFR identified as a potential target of compound **3**.

Keywords: fungus/*Cordyceps*-colonizing fungus/structure elucidation/cytotoxicity/network pharmacology/molecular docking

Contents **Page**

General experimental procedure ... 2

ECD calculation methods ... 2

MTS assay ... 2

Figure S1. ^{1}H NMR spectrum of pseudogymnone A (1; 500 MHz, $CDCl_3$) ... 4

Figure S2. ^{13}C NMR spectrum of pseudogymnone A (1; 125 MHz, $CDCl_3$) ... 5

Figure S3. HMBC spectrum of pseudogymnone A (1; 500 MHz, $CDCl_3$) ... 6

Figure S4. HMQC spectrum of pseudogymnone A (1; 500 MHz, $CDCl_3$) ... 7

Figure S5. ^{1}H-^{1}H COSY spectrum of pseudogymnone A (1; 400 MHz, $CDCl_3$) ... 8

Figure S6. NOESY spectrum of pseudogymnone A (1; 500 MHz, $CDCl_3$) ... 9

Figure S7. ^{1}H NMR spectrum of penijanthinone C (2; 600 MHz, $CDCl_3$) ... 10

Figure S8. ^{13}C NMR spectrum of penijanthinone C (2; 125 MHz, $CDCl_3$) ... 11

Figure S9. HMQC spectrum of penijanthinone C (2; 600 MHz, $CDCl_3$) ... 12

Figure S10. ^{1}H-^{1}H COSY spectrum of penijanthinone C (2; 600 MHz, $CDCl_3$) ... 13

Figure S11. HMBC spectrum of penijanthinone C (2; 600 MHz, $CDCl_3$) ... 14

Table S1. ECD conformers of pseudogymnone A (1) ... 15

Table S2. ECD conformers of penijanthinone C (2) ... 22

General experimental procedure

Optical rotations were measured on an Anton Paar MCP 200 Automatic Polarimeter (Anton Paar, Graz, Austria) and UV data were obtained on a Thermo Genesys-10S UV/Vis spectrophotometer (Thermo Fisher Scientific, Waltham, MA, USA). The ECD spectra were measured by JASCO J-815 spectropolarimeter (JASCO, Tsukuba, Japan). IR data were recorded using a Nicolet IS5 FT-IR spectrophotometer (Thermo Fisher Scientific, Waltham, MA, USA). ^{1}H and ^{13}C NMR data were acquired with Bruker Avance-500 spectrometer (Bruker, Bremen, Germany) using solvent signals ($CDCl_3$; δ_H 7.26/δ_C 77.7) as references. ESIMS data were recorded on a Bruker Esquire 3000^{plus} spectrometer (Bruker, Bremen, Germany), and HRESIMS data were obtained using Bruker APEX III 7.0 T and APEX II FT-ICR spectrometers (Bruker, Bremen, Germany), respectively.

ECD calculation methods

Conformational analysis within an energy window of 3.0 kcal/mol was performed by using the OPLS3 molecular mechanics force field via the MacroModel panel of Maestro 10.2. The conformers were then further optimized with the software package Gaussian 09 at the B3LYP/6-311G (d,p) level. Then the 60 lowest electronic transitions for the obtained conformers were calculated using time-dependent density functional theory (TD-DFT) methods at the CAM-B3LYP/6-311G (d,p) level. ECD spectra of the conformers were simulated using a Gaussian function. The overall theoretical ECD spectra were obtained according to the Boltzmann weighting of each conformers.

MTS assay

The assay was run in triplicate. In a 96-well plate, each well was plated with (2–5) × 10^3 cells (depending on the cell multiplication rate). After cell attachment overnight, the medium was removed, and each well was treated with 100 μL of medium contain-ing 0.1% DMSO, or appropriate

concentrations of the test compounds and the positive control paclitaxel (Sigma) (100 mM as stock solution of a compound in DMSO and se-rial dilutions; the test compounds showed good solubility in DMSO and did not precip-itate when added to the cells). The plate was incubated for 72 h at 37°C in a humidified, 5% CO_2 atmosphere. Proliferation was assessed by adding 20 μL of MTS (Promega) to each well in the dark, followed by a 90 min incubation at 37°C. The assay plate was read at 490 nm using a microplate reader. IC_{50} values were determined via variable-slope nonlinear regression of fitted sigmoidal dose-response curves (GraphPad Prism 9.0). All cancer cells A549 (ATCC CCL-185), CNE1-LMP1 (Cancer Hospital, Chinese Academy of Medical Sciences), A375 (ATCC CRL-1619), MCF-7 (ATCC HTB-22), MGC (1101HUM-PUMC000660), EC109 (1101HUM-PUMC000246), PANC-1 (ATCC CRL-1469), Hep3B-2 (ATCC HB-8064), and HaCaT (1101HUM-PUMC000373) were obtained from Cancer Research Institute, Xiangya School of Medicine, Central South University.

Figure S1. ^{1}H NMR spectrum of pseudogymnone A (**1**; 500 MHz, $CDCl_3$)

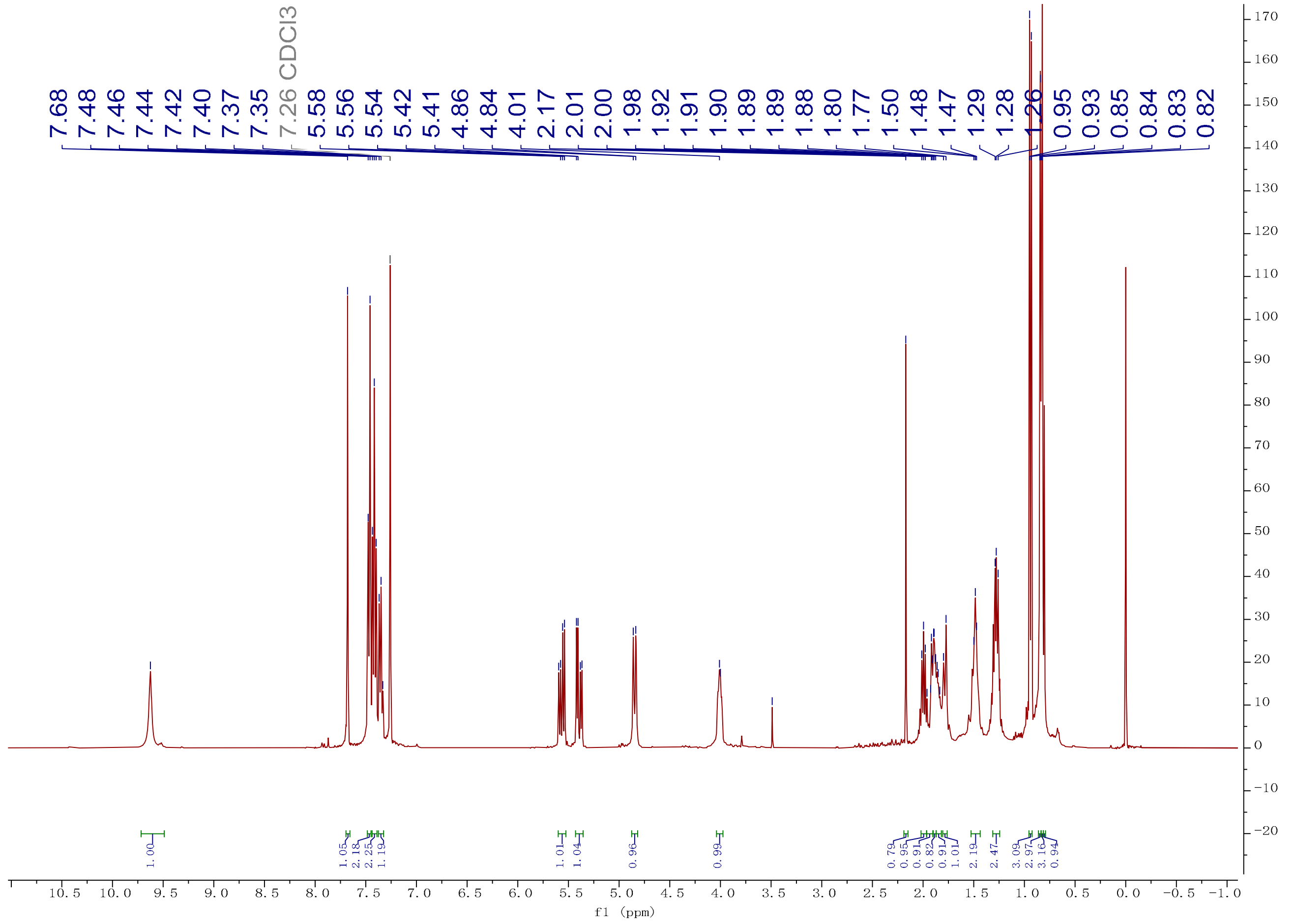

Figure S2. ^{13}C NMR spectrum of pseudogymnone A (**1**; 125 MHz, $CDCl_3$)

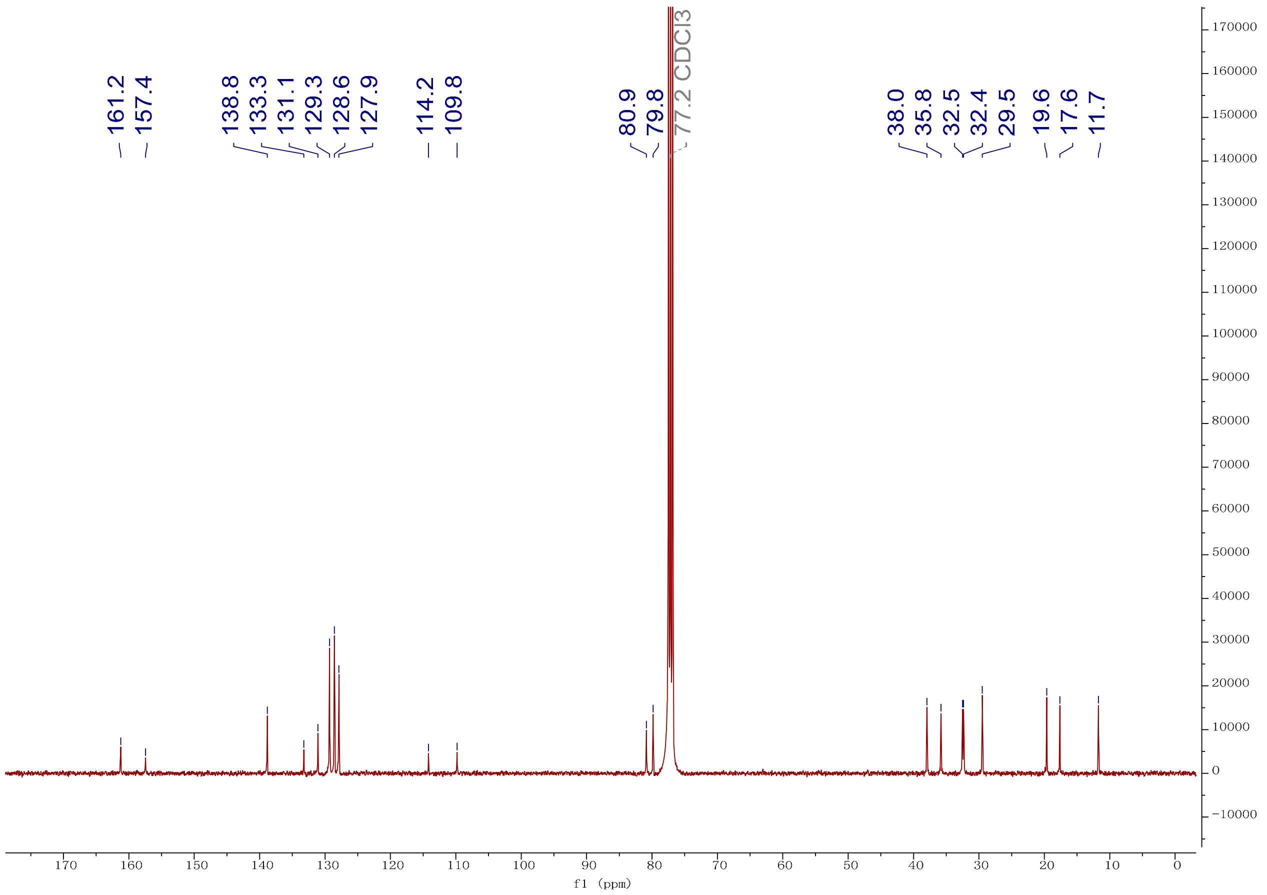

Figure S3. HMBC spectrum of pseudogymnone A (**1**; 500 MHz, $CDCl_3$)

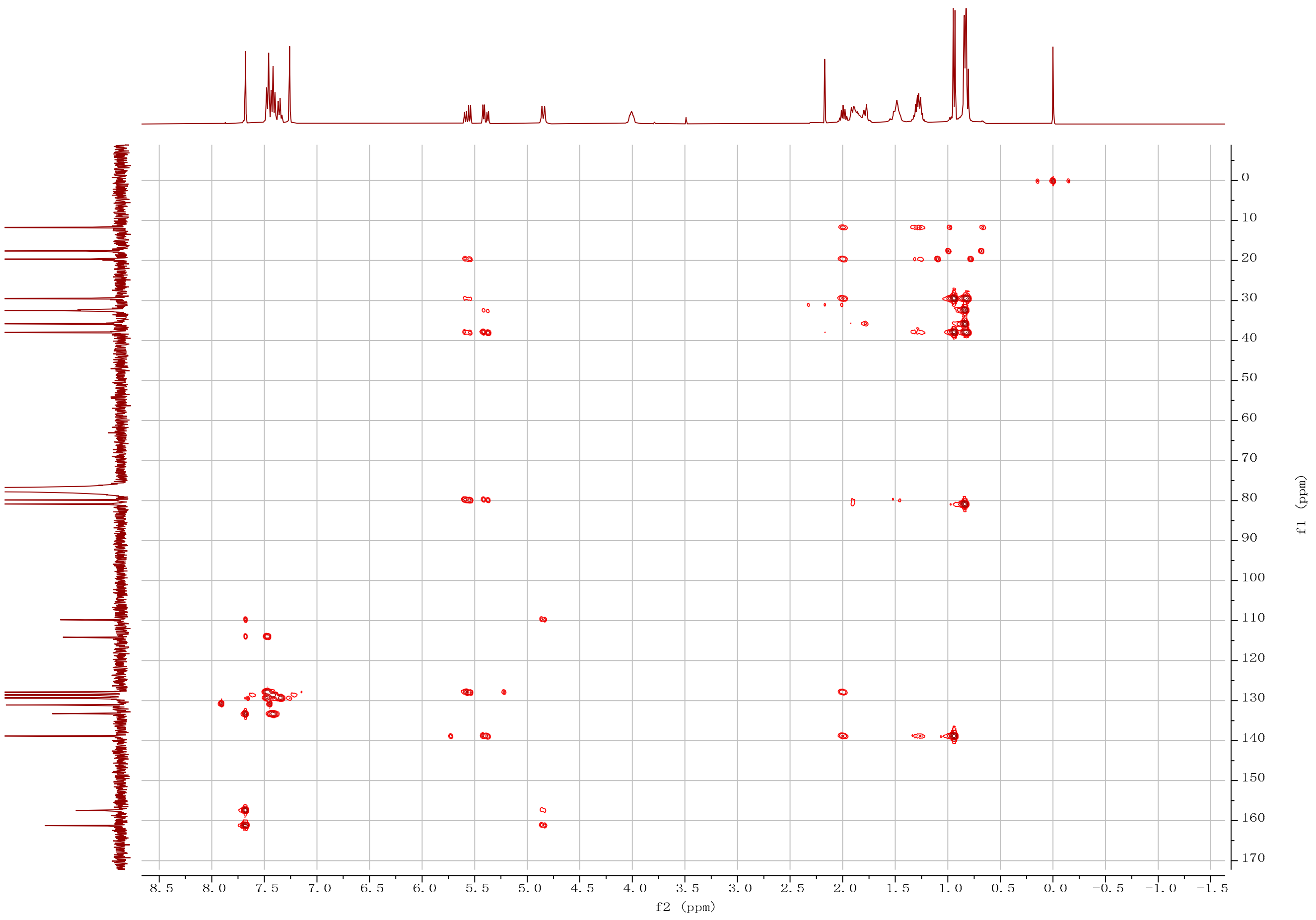

Figure S4. HMQC spectrum of pseudogymnone A (**1**; 500 MHz, $CDCl_3$)

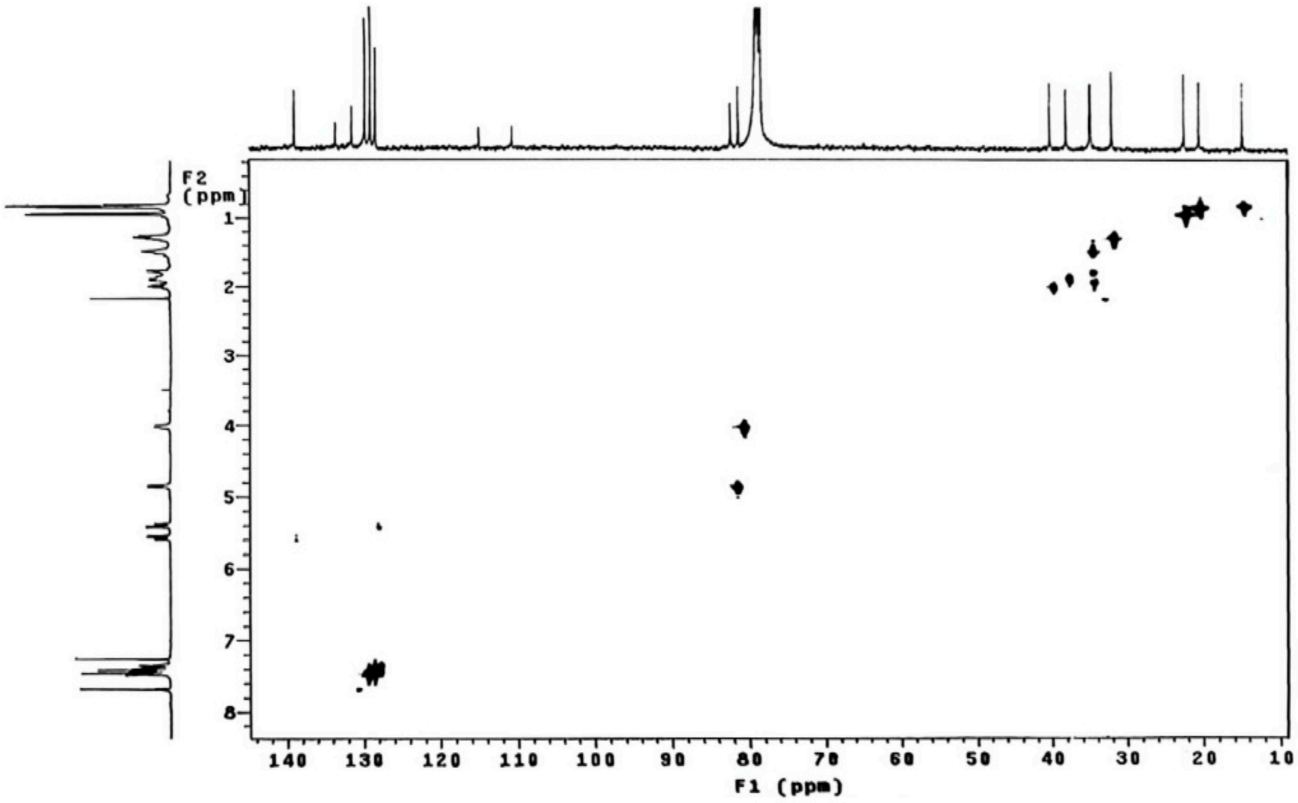

Figure S5. ^{1}H-^{1}H COSY spectrum of pseudogymnone A (**1**; 400 MHz, $CDCl_3$)

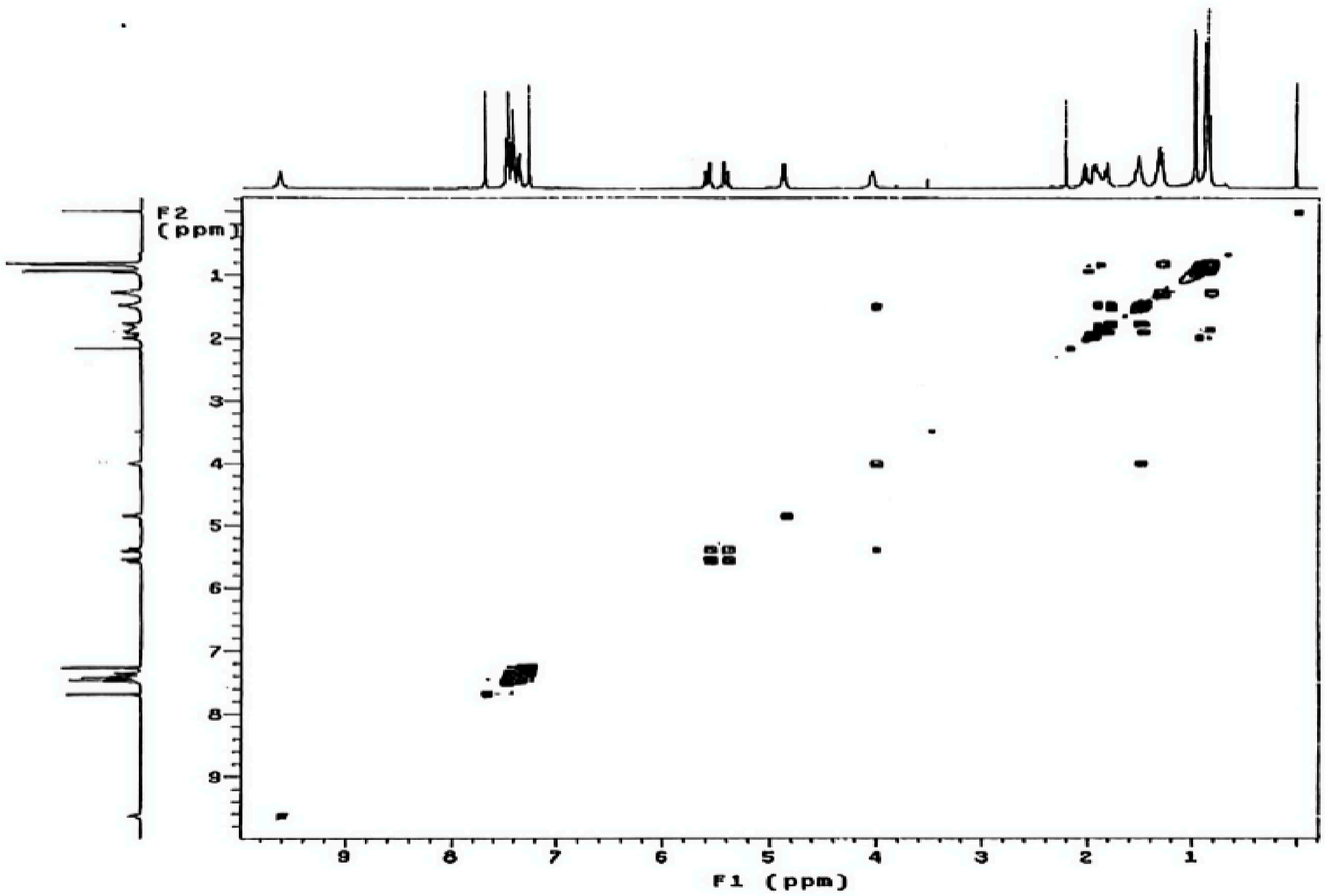

Figure S6. NOESY spectrum of pseudogymnone A (**1**; 500 MHz, $CDCl_3$)

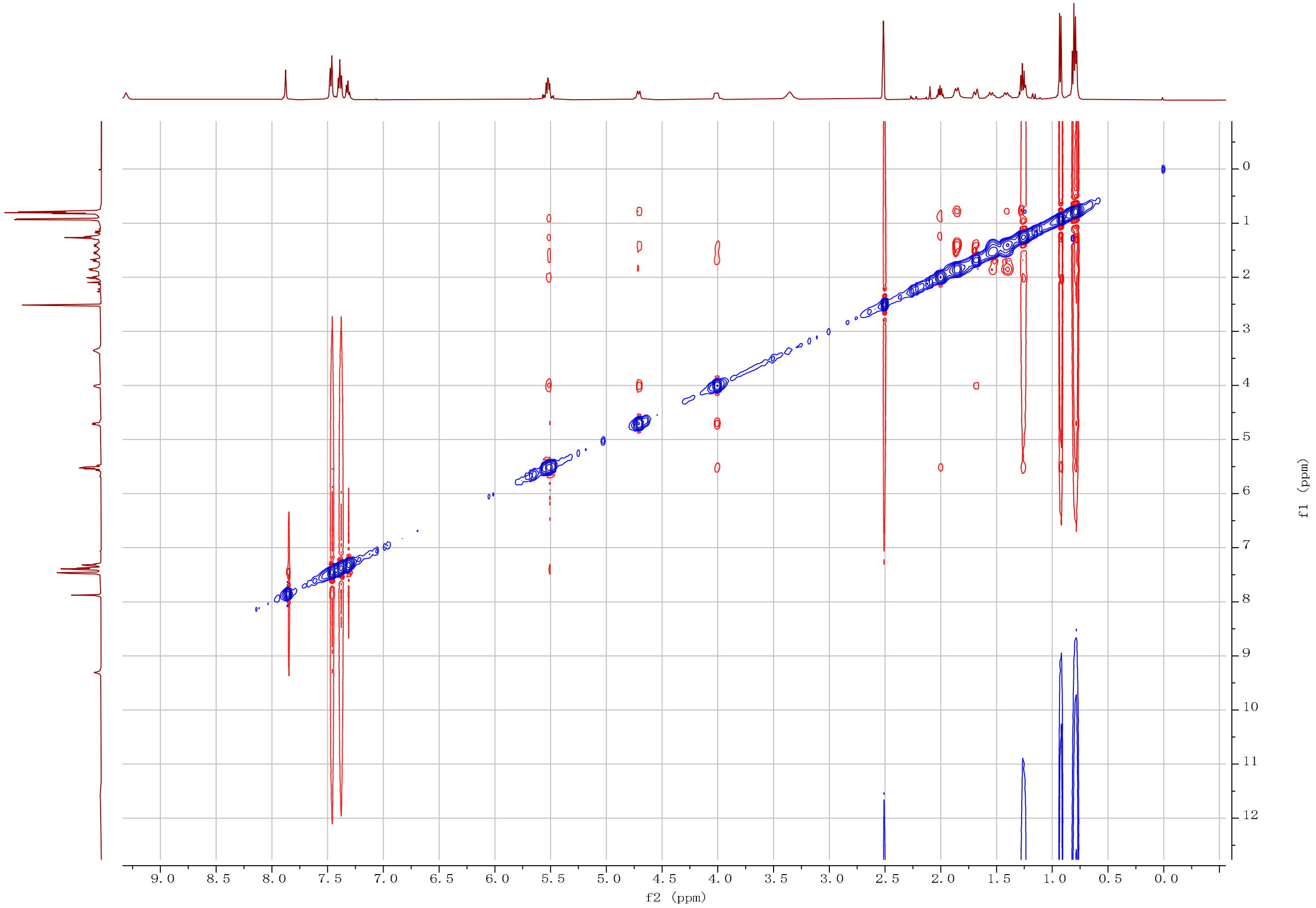

Figure S7. ^{1}H NMR spectrum of penijanthinone C (**2**; 600 MHz, $CDCl_3$)

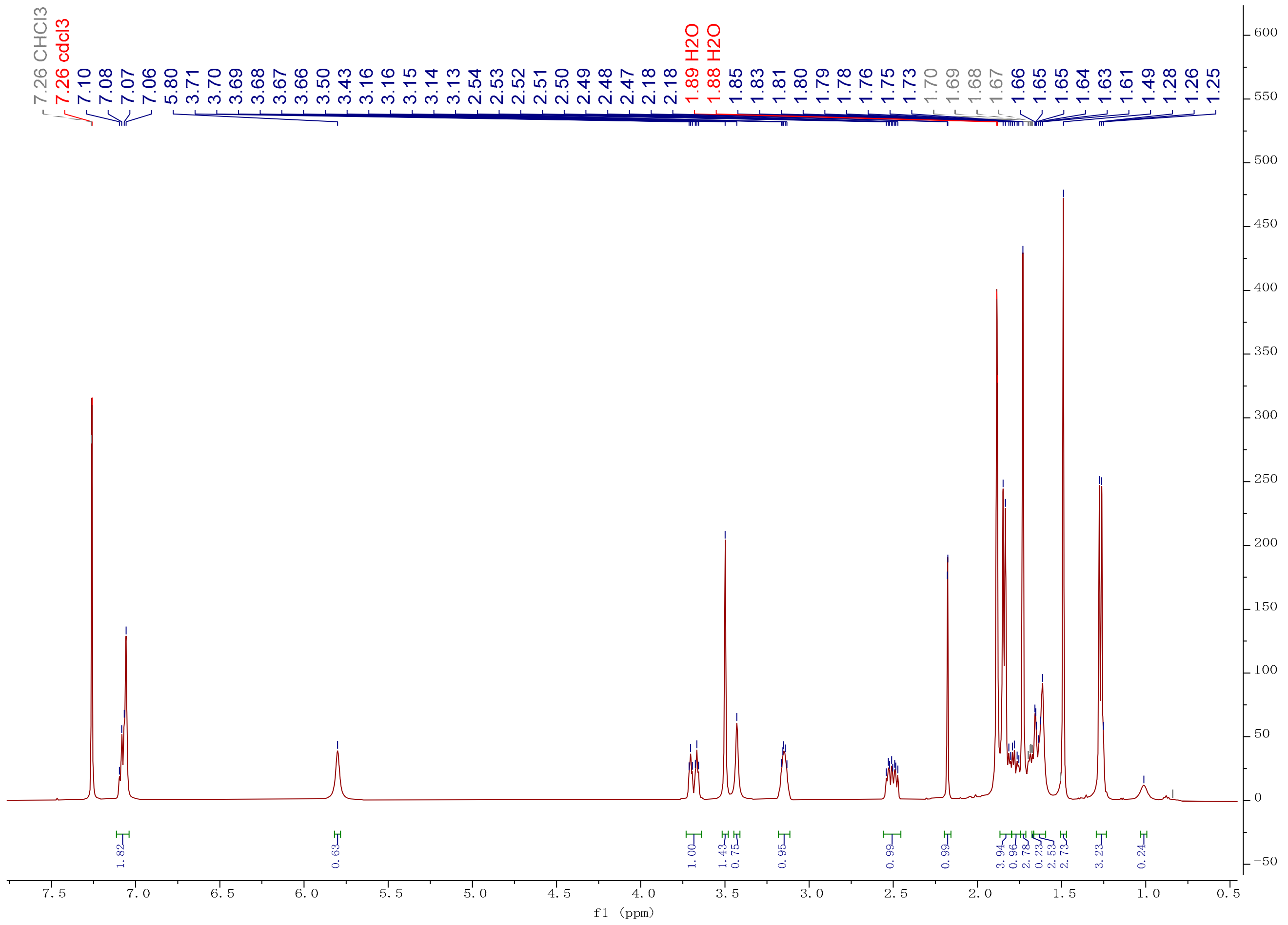

Figure S8. ^{13}C NMR spectrum of penijanthinone C (**2**; 125 MHz, $CDCl_3$)

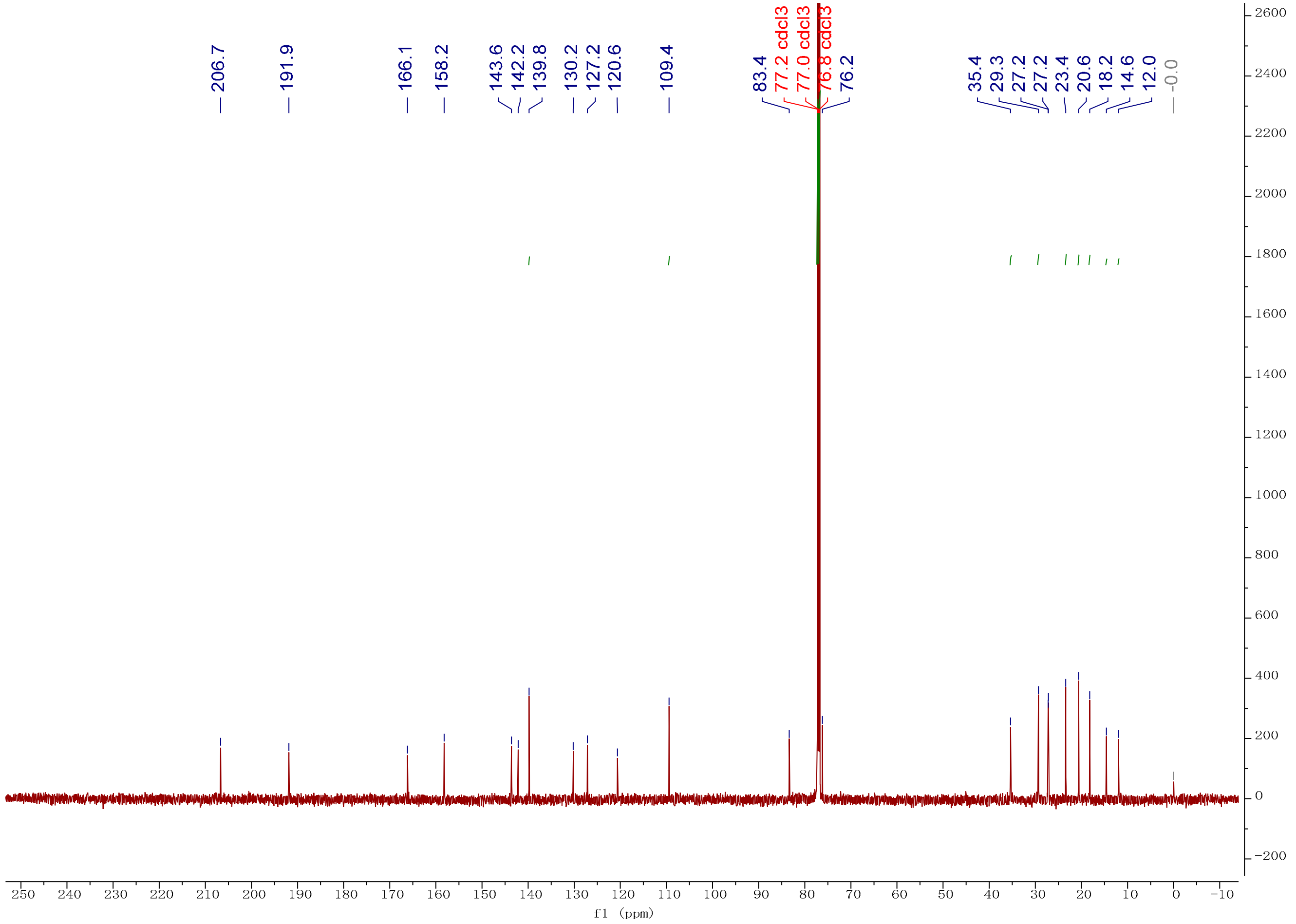

Figure S9. HMQC spectrum of penijanthinone C (**2**; 600 MHz, $CDCl_3$)

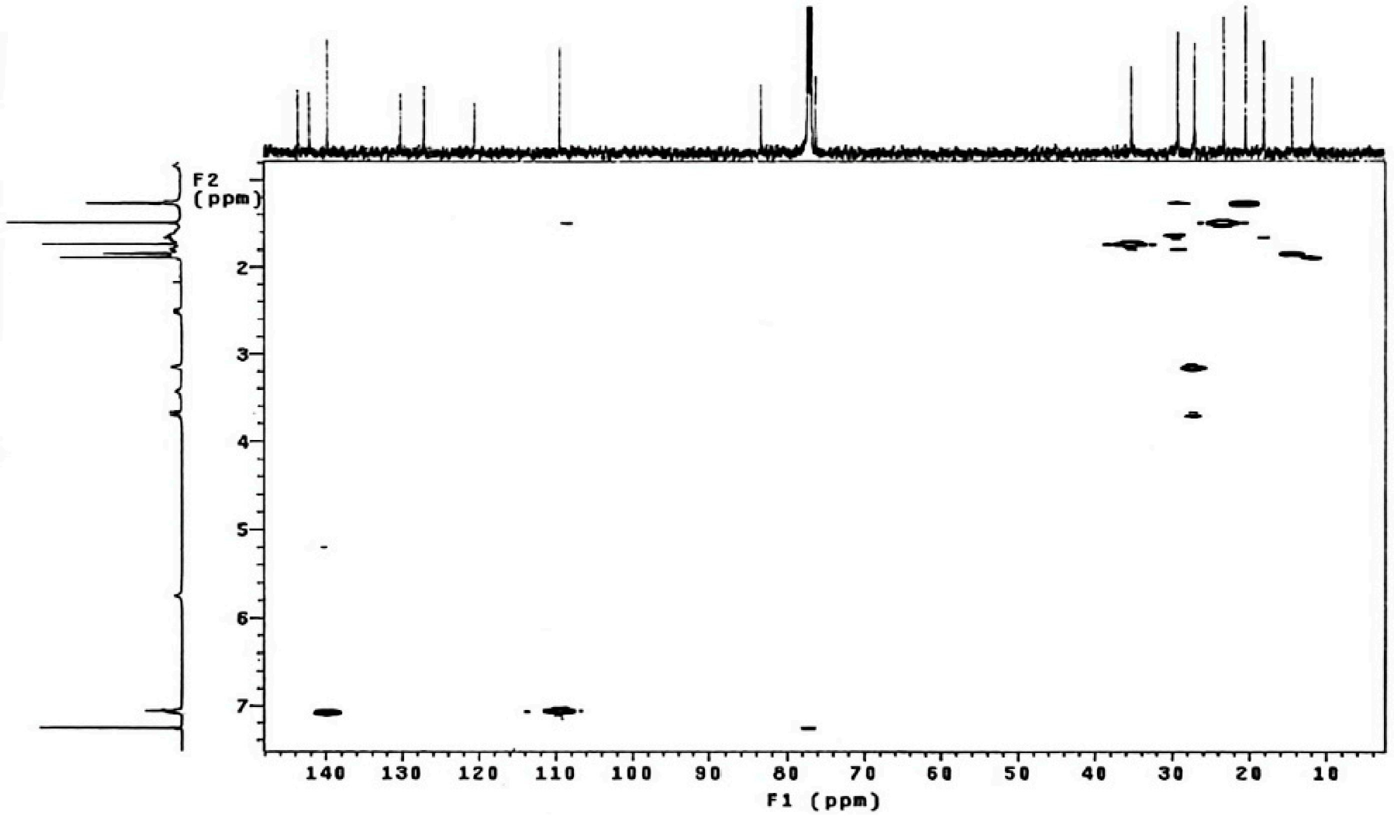

Figure S10. ^{1}H-^{1}H COSY spectrum of penijanthinone C (**2**; 600 MHz, $CDCl_3$)

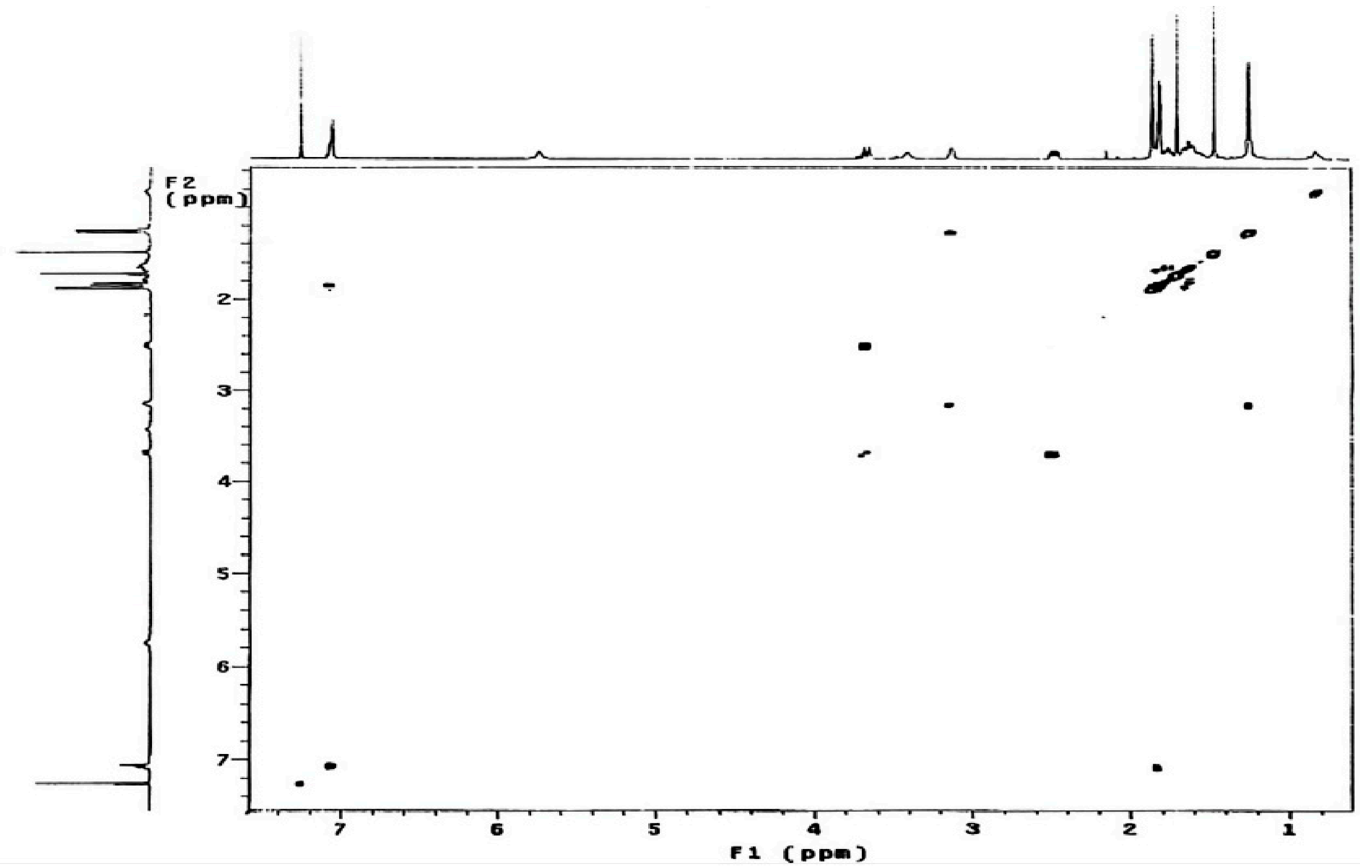

Figure S11. HMBC spectrum of penijanthinone C (**2**; 600 MHz, $CDCl_3$)

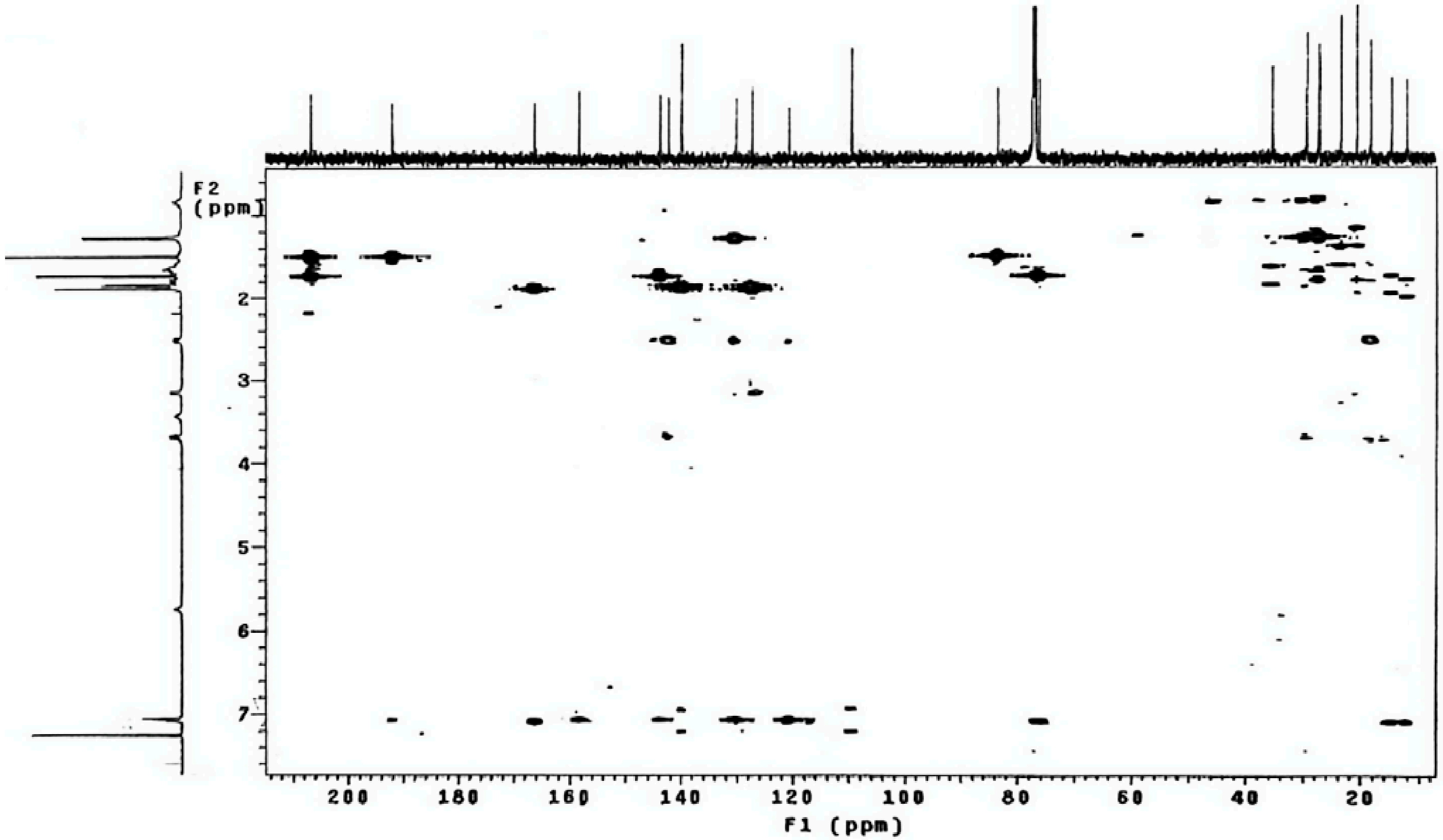

Table S1. ECD conformers of pseudogymnone A (**1**)

Configuration	Population(%)	Conformers	Configuration	Population(%)	Conformers
**1a**	17.93	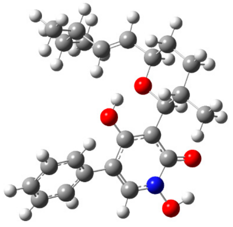	**1b**	15.75	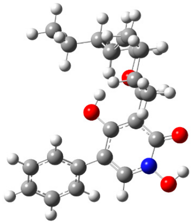
	12.85	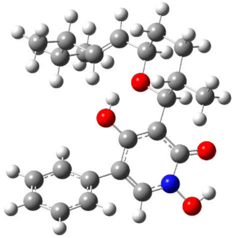		11.24	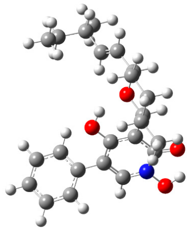
	2.68	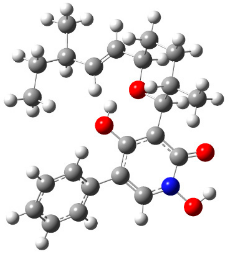		2.27	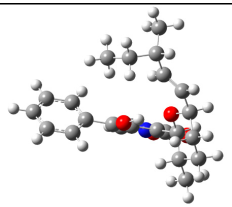

	1.56	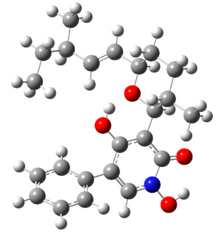		1.35	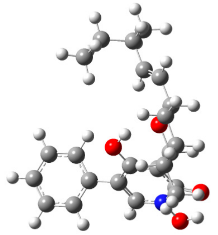
	1.79	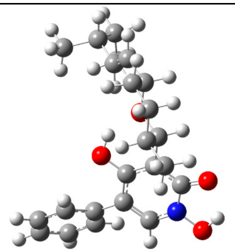		6.24	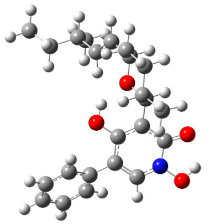
	6.66	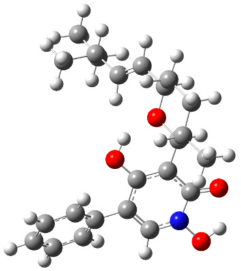		14.17	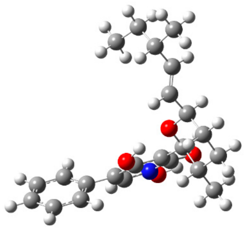
	11.84	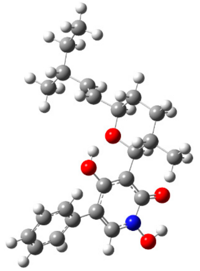		4.48	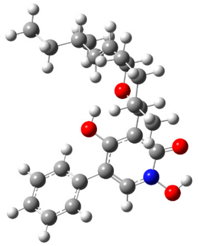

	1.29	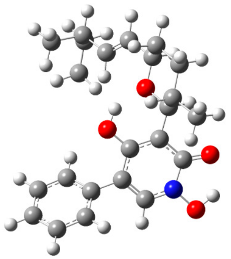		12.48	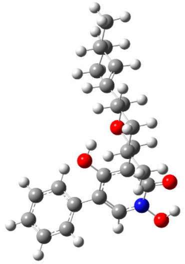
	4.85	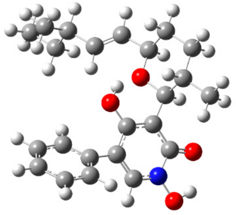		1.59	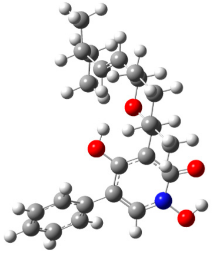
	9.97	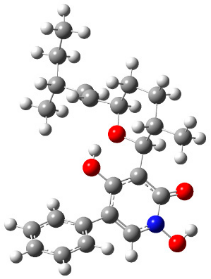		1.19	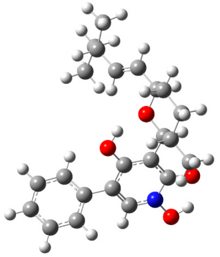

	5.04	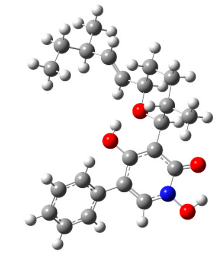		1.21	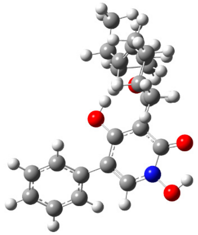
	4.31	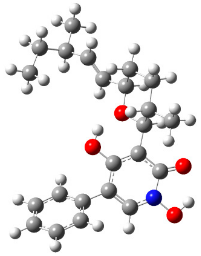		4.81	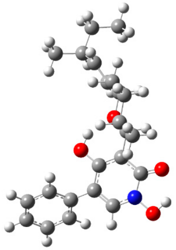
	2.18	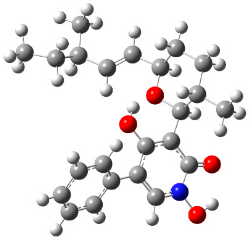		4.48	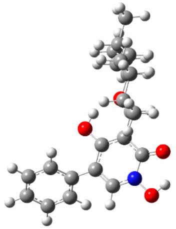

	4.74	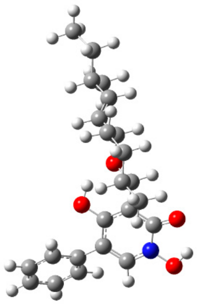		1.95	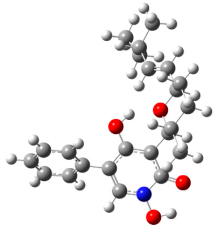
	1.68	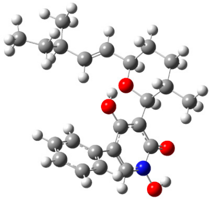		1.19	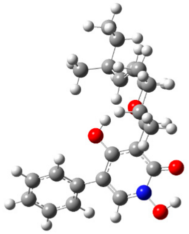
	4.04	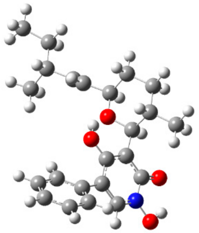		4.12	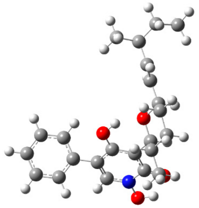
	2.09	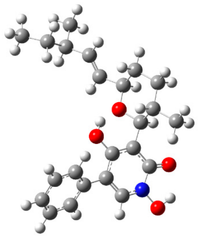		3.89	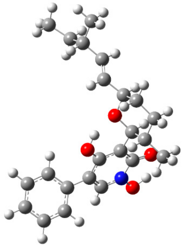

	1.76	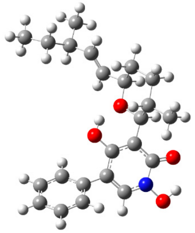		1.42	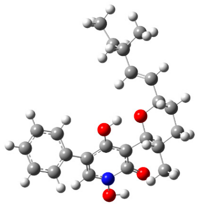
	1.47	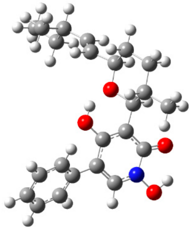		1.88	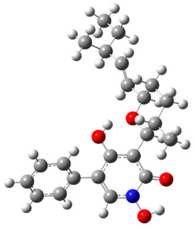
	1.28	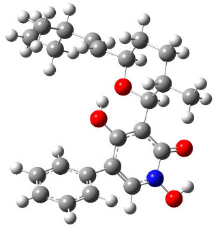		1.42	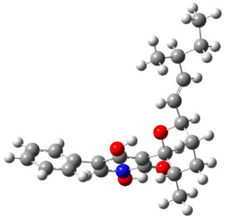
				1.61	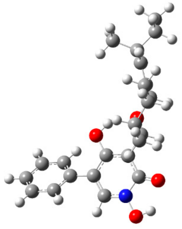

				1.24	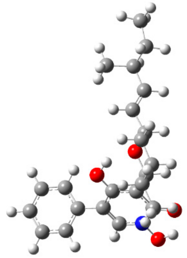

Table S2. ECD conformers of penijanthinone C (**2**)

Configuration	Population(%)	Conformers
**2**	1.46	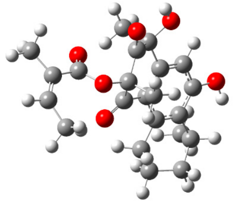
	4.2	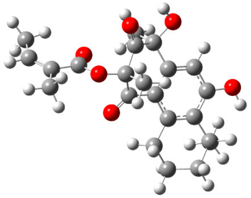
	3.94	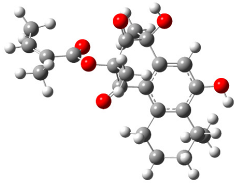

	1.07	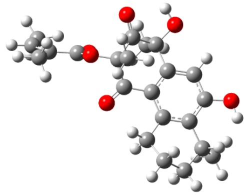
	4.73	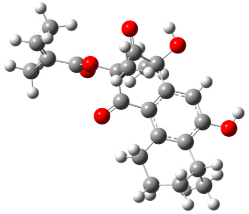
	12.55	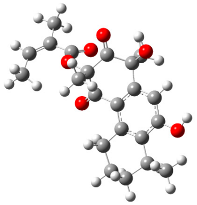
	1.19	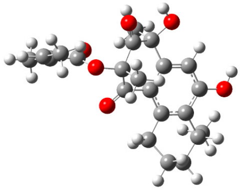

	3.2	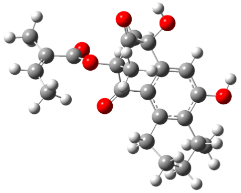
	34.22	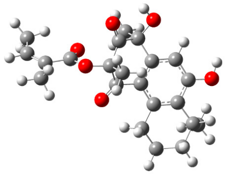
	33.44	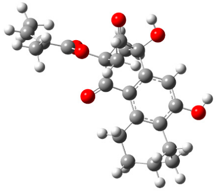
